# *In vivo* TSPO imaging in patients with multiple sclerosis: a brain PET study with [^18^F]FEDAA1106

**DOI:** 10.1186/2191-219X-3-30

**Published:** 2013-04-24

**Authors:** Akihiro Takano, Fredrik Piehl, Jan Hillert, Andrea Varrone, Sangram Nag, Balázs Gulyás, Per Stenkrona, Victor L Villemagne, Christopher C Rowe, Richard Macdonell, Nabil Al Tawil, Thomas Kucinski, Torsten Zimmermann, Marcus Schultze-Mosgau, Andrea Thiele, Anja Hoffmann, Christer Halldin

**Affiliations:** 1 Center for Psychiatric Research, Department of Clinical Neuroscience, Karolinska Institutet, Stockholm SE-171 77, Sweden; 2 Department of Clinical Neuroscience, Karolinska Institutet, Stockholm SE-171 77, Sweden; 3 Department of Nuclear Medicine and Centre for PET, Austin Health, Heidelberg 3084, Australia; 4 Department of Neurology, Austin Health, Heidelberg 3084, Australia; 5 Karolinska Trial Alliance, Karolinska University Hospital, Stockholm SE-141 86, Sweden; 6 Bayer Health Care AG, Berlin, Germany

**Keywords:** Multiple sclerosis, Positron emission tomography, TSPO imaging

## Abstract

**Background:**

The activation of microglia, in general, and the upregulation of the translocator protein (18 kDa) (TSPO) system, in particular, are key features of neuroinflammation, of which the *in vivo* visualization and quantitative assessment are still challenging due to the lack of appropriate molecular imaging biomarkers. Recent positron emission tomography (PET) studies using TSPO radioligands such as [^11^C]PK11195 and [^11^C]PBR28 have indicated the usefulness of these PET biomarkers in patients with neuroinflammatory diseases, including multiple sclerosis (MS). [^18^F]FEDAA1106 is a recently developed PET radioligand for the *in vivo* quantification of TSPO. In the present study, we aimed at investigating the diagnostic usefulness of [^18^F]FEDAA1106 in patients with MS.

**Methods:**

Nine patients (three on the interferon beta therapy and six without immunomodulatory therapy; seven females/two males; age 34.2 ± 9.1 years old) with relapsing-remitting MS in acute relapse and with gadolinium (Gd)-enhancing lesion(s) in the magnetic resonance imaging (MRI) scans and five healthy controls (four females/one male, age 38.0 ± 9.7 years old) were investigated in this study. Genetic information about the TSPO binding could not be obtained because knowledge about the importance of genetic background for TSPO binding was not available at the time the study was performed. Dynamic PET measurements were performed using an ECAT EXACT HR system (CTI/Siemens, Knoxville, TN, USA) for a total of 150 min, with a 30-min break after the injection of 153.4 ± 10.2 MBq of [^18^F]FEDAA1106. Metabolite-corrected arterial plasma samples were used to calculate the input function. PET data were analyzed in the following ways: (1) region-of-interest analysis for cortical and subcortical regions was performed using a two-tissue compartment kinetic model in order to estimate binding potentials (BP_ND_) and distribution volume (VT), (2) the feasibility of the estimation of BP_ND_ and VT was investigated for MS lesions, and (3) VT parametric images by a Logan plot and standard uptake value (SUV) images were visually compared with the corresponding MRI, focusing on MRI-identified MS lesions.

**Results:**

There were no significant differences in the BP_ND_ or VT values between patients with MS and healthy controls. Robust BP_ND_ and VT values could not be obtained for most MS lesions due to noisy time-activity curves. Visual inspection of VT and SUV images in all nine patients did not reveal high uptake of the radioligand inside and beyond MRI-identified active MS lesions with the exception of one Gd-enhanced MS lesion in the whole patient population.

**Conclusions:**

In our study, [^18^F]FEDAA1106 as a PET radioligand could neither differentiate patients with MS from healthy controls nor detect active plaques in the brain of MS patients. Stratification with respect to genetics and binder status might help to uncover the differences between the groups, which could not be detected here.

**Trial registration:**

ClinicalTrials.gov, NCT01031199

## Background

Multiple sclerosis (MS) is characterized by neuroinflammation, demyelination, and axonal loss in the brain. The activation of microglia cells is one of the main components of neuroinflammation. The expression of the translocator protein (TSPO) is reported to increase in activated microglia as well as infiltrating macrophages and reactive astrocytes in MS [1]. The evaluation of the TSPO system may provide us with information about the activity of the disease and its progression. Recently, TSPO has been visualized and quantified *in vivo* by positron emission tomography (PET) with various radioligands. PET studies, using [^11^C]PK11195, have reported increased binding in areas of focal MS lesions defined by MRI scans, especially Gd-enhanced lesions [2]. [^11^C]PBR28 displayed significantly higher white matter-gray matter ratios in MS patients than in healthy controls as well as increased radioligand binding in areas beyond focal MRI-defined MS lesions [3]. [^11^C]vinpocetine exhibited local radioligand uptakes in the vicinity of the MRI-identified MS lesions [4].

[^18^F]FEDAA1106 is a recently developed PET radioligand for the quantification of TSPO *in vivo*. Compared with PBR28 and PK11195, FEDAA1106 has higher affinity to TSPO (Ki = 0.078 nM) [5]. ^18^F radioligands have an advantage over ^11^C radioligands with respect to the longer half-life (109 vs 20 min), which makes transportation of the ligands to PET centers, without cyclotrons, possible. In the present study, we have investigated the diagnostic potential of [^18^F]FEDAA1106 to detect MS lesions and to differentiate between patients with MS and healthy volunteers.

## Methods

### Radiochemistry

[^18^F]FEDAA1106 was synthesized, as described previously [6]. The mean injected radioactivity was 153.4 ± 10.2 MBq. The mass injected was less than 2.7 μg.

The injection of the radioligand was performed as a bolus over 30 s via an inserted intravenous line, and the cannula was flushed with 10 mL of saline.

### Subjects

The study was conducted in line with the Helsinki Declaration and approved by the Independent Ethics Committee and the Radiation Safety Committee of the Karolinska University Hospital as well as the Swedish Medical Products Agency. The study was registered at www.ClinicalTrials.gov. Nine patients (seven females/two males; age 34.2 ± 9.1 years old) with relapsing-remitting MS in acute relapse were included in this study; six of them were drug-naive, and three patients were on beta-interferon treatment at the time of the PET measurements. All the patients fulfilled the following criteria: clinical signs of a relapse within a maximum of 21 days before screening, at least two T2 lesions at different locations and at least one Gd-enhanced lesion in the MRI screening and a score between 0 and 5 points on the Kurtzke expanded disability status scale. Five healthy volunteers (four females/one male, age 38.0 ± 9.7 years old) were included in this study. Subjects who had other chronic diseases or conditions or who used concomitant medication that could interfere with measurements, such as treatment with benzodiazepines, were excluded. Likewise, any unstable medical conditions as well as patients with either evidence or history of a severe psychiatric disorder were judged ineligible for this study. Healthy volunteers with abnormal MRI findings for their ages were also excluded. Informed written consent was obtained from all the subjects. The time span between the MRI screening and the PET scan was 3.6 ±1.4 days (1 to 6 days) for MS patients and 9.8 ± 3.9 days (5 to 15 days) for healthy volunteers.

### MRI measurements

MRI T1-weighted images were acquired using the 1.5 T GE Signa system (GE Medical Systems, Milwaukee, WI, USA). T1-weighted, T2-weighted, and fluid-attenuated inversion recovery (FLAIR) images were acquired for both MS patients and healthy controls. Gd-enhanced T1 images were also acquired for MS patients.

### PET measurements with arterial blood sampling

PET measurements were performed on an ECAT Exact HR 47 PET system (CTI/Siemens, Knoxville, TN, USA) operated in 3D mode. The scanner’s three-ring detector block architecture provides a 15-cm-wide field of view. The transversal resolution in the reconstructed image is about 3.8 mm full width at half maximum (FWHM), and the axial resolution is 3.125 mm [7]. Attenuation correction was obtained with three rotating ^68^Ge line sources. Emission data of [^18^F]FEDAA1106 were acquired over 150 min with a 30-min break between 60 and 90 min after the radioligand injection. The frame times were 20 s × 6 frames, 1 min × 4 frames, 3 min × 6 frames, 6 min × 6 frames in the first part, and 6 min × 10 frames in the second part. Images were reconstructed using the standard filtered back projection with a 2-mm Hanning filter.

A catheter was inserted in the radial artery, and arterial blood was collected continuously for 5 min using an automated blood-sampling system at a speed of 5 mL/min (ABSS; Allog AB, Mariefred, Sweden). Blood samples (4 mL) were drawn at 2.5, 9, 20, 30, 40, 60, 90, 120, and 150 min for blood and plasma radioactivity and metabolite correction.

### Metabolite analysis

A reversed-phase high-performance liquid chromatography (HPLC) method was used to determine the percentages of radioactivity in plasma that corresponded to both unchanged [^18^F]FEDAA1106 and its radioactive metabolites during the course of the PET measurement. Plasma (0.5 mL), obtained after centrifugation of blood at 2,000×*g* for 2 min, was mixed with acetonitrile (0.7 mL). After additional centrifugation of the acetonitrile-plasma mixture (1.1 mL) at 2,000×*g* for 2 more min, the supernatant was measured in a NaI well counter and then analyzed by radio-HPLC.

The radio-HPLC system consisted of an interface module (D-7000; Hitachi, Chiyoda-ku, Japan), an L-7100 pump (Hitachi), an injector (Rheodyne model 7125 with a 1.0-mL loop; IDEX Corporation, Oak Harbor, WA, USA), and an absorbance detector (L-7400; 254 nm; Hitachi) in series with a radiation detector (Radiomatic 150TR; Packard, Meriden, CT, USA) equipped with a PET flow cell (600 μL cell). A μ-Bondapak-C18 column (300 mm × 7.8 mm, 10 μm; Waters, Milford, MA, USA) was used for metabolite analysis. The following gradient settings were used with a flow rate of 6.0 mL/min: solvents - acetonitrile (A) and phosphoric acid (10 mM) (B); time 0 min 25 (A) and 75 (B); time 4.5 min 80 (A) and 20 (B); time 8.0 min 30 (A) and 70 (B); and time 10.0 min 25 (A) and 75 (B).

### Image data analysis

Data analyses were performed focusing on the following three main points: kinetic compartment model analysis for brain anatomical regions, feasibility of estimation of outcome measures for MS lesions, and visual inspection of PET images. Group differences between patients with MS and control subjects were intended to be investigated by the evaluation of brain anatomical regions. As Gd (+) and/or T2/FLAIR high-intensity lesions were considered to reflect on inflammatory demyelization, and the increase of TSPO binding was reported in such areas in previous PET studies [2,8], the feasibility of a quantitative evaluation of MRI-defined MS lesions was explored. In one of the previous PET studies [3], high TSPO binding was reported in the areas beyond the MRI-defined MS lesions. Additionally, visual inspection of the PET images was performed in order to detect such changes.

### Kinetic analysis for brain anatomical regions

PET images of the first and second parts were coregistered to the T1-weighted MRI using an SPM5 software (Wellcome Department of Imaging Neuroscience, London, UK). Regions of interest were manually delineated on the individual T1-weighted MRIs. The following regions were defined: frontal cortex, orbitofrontal cortex, lateral temporal cortex, insular cortex, parietal cortex, occipital cortex, anterior cingulate cortex, posterior cingulate cortex, caudate, putamen, thalamus, hippocampus, cerebellum, midbrain, and pons. A VOI for the whole grey matter was obtained from the segmented MRI. Time-activity curves for regions of interest (ROIs) were generated by applying the ROIs to the corresponding dynamic PET data.

In this study, binding potential (BP_ND_) and total distribution volume (VT), as outcome measures for [^18^F]FEDAA1106 binding and brain distribution, were calculated using a two-tissue compartment (2TC) model [9]. Comparisons between patients with MS and control subjects were made for BP_ND_ and VT values, respectively. In addition, a medication-dependent stratification of the MS group was made.

### Feasibility of estimation of BP_ND_ and VT for MS lesions

In the MRI brain images of MS patients, ROIs were delineated on MRI-defined MS lesions revealed by FLAIR and T2 and T1 Gd-enhanced scans. We focused on the MRI-defined MS lesions of the following two groups: Gd-enhanced MS lesions (total 49 lesions) and non-Gd-enhanced-but-T2/FLAIR-high-intensity MS lesions (total 159 lesions).

Time-activity curves for each MS lesion as well as for summed subgroups were generated by applying the ROIs to the dynamic PET data. BP_ND_ and VT were calculated by 2TC.

### Visual inspection of PET images

For visual inspection of PET images, VT and standard uptake value (SUV) images were generated. VT parametric images were generated using the Logan plot (T* = 48 min) [10]. SUV images were generated from the 90-to-150-min PET images.

The parametric images were visually compared with MRI images, focusing on MRI-defined MS lesions.

Kinetic analysis and generation of parametric images were performed using the software package PMOD (PMOD 3.0, PMOD Group, Zurich, Switzerland).

## Results and discussion

### Results

#### Kinetic analysis for brain regions

**Figure 1:**
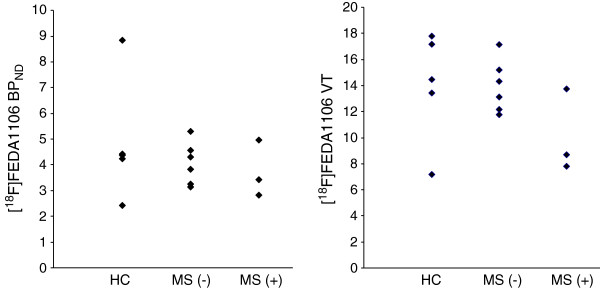
**Comparison of BP**_**ND **_**(left) and VT (right) of [**^**18**^**F]FEDAA1106 in whole gray matter among MS patients.** Six without treatment (MS(−)), three with treatment (MS(+)), and five healthy controls (HC). There were no significant differences of BP_ND_ or VT.

When all MS patients were compared with control subjects, there was no significant difference of BP_ND_ or VT in any of the evaluated regions. Furthermore, when the MS patients were divided based on the drug treatment, there was no significant difference of BP_ND_ or VT among the three groups: MS without treatment, MS with treatment, and normal control (Figure 1; Tables 1 and 2).

**Table 1:** **Regional BP**_**ND **_**values among MS patients and healthy controls**

**Region**	**Six MS naive**			**Three MS treatment (+)**			**Five HC**		
	**Mean**	**Min**	**Max**	**Mean**	**Min**	**Max**	**Mean**	**Min**	**Max**
Whole gray matter	4.1	3.2	5.3	3.7	2.8	5.0	4.9	2.4	8.9
ACC	4.5	2.7	7.1	3.4	2.1	5.5	5.8	2.5	13.5
Caudate	3.7	2.1	4.7	3.5	2.7	4.8	5.1	2.1	9.5
Putamen	4.1	3.2	5.2	3.6	2.2	4.9	4.3	2.1	8.2
Thalamus	4.5	3.4	5.9	5.2	2.8	8.8	6.1	2.4	13.9
Cerebellum	4.2	3.2	5.1	3.7	2.9	4.9	4.9	2.4	9.1
Frontal cortex	4.2	3.3	5.6	4.0	2.9	5.4	5.1	2.6	8.9
Hippocampus	4.5	3.1	6.9	5.1	2.0	9.2	5.2	3.1	9.8
Insular cortex	4.0	3.2	4.9	4.1	2.9	5.3	4.6	2.2	8.5
Lateral temporal cortex	4.3	3.1	5.6	3.9	2.9	5.0	5.1	2.7	9.4
Midbrain	6.4	3.0	14.0	6.6	2.5	13.0	5.5	2.9	11.7
Occipital cortex	3.8	3.0	4.6	3.4	2.5	4.5	4.4	2.5	7.0
Orbitofrontal cortex	5.4	3.2	12.2	4.0	2.8	5.3	5.1	2.8	9.1
Parietal cortex	4.0	3.2	5.2	3.7	2.9	4.5	4.7	2.6	7.7
PCC	4.3	2.5	6.3	4.4	2.4	8.0	5.4	2.7	8.4
Pons	4.5	3.4	6.0	4.4	2.4	8.0	6.0	3.9	11.7

There was no significant difference of the BP_ND_ values in any region among the three groups.

**Table 2:** Regional VT values among MS patients and healthy controls

**Region**	**Six MS naive**			**Three MS treatment (+)**			**Five HC**		
	**Mean**	**Min**	**Max**	**Mean**	**Min**	**Max**	**Mean**	**Min**	**Max**
Whole gray matter	14.0	11.8	17.2	10.1	7.8	13.8	14.0	7.2	17.8
ACC	14.8	11.5	19.2	9.7	7.3	14.4	13.8	5.9	19.4
Caudate	12.8	10.5	16.2	10.2	8.0	14.1	13.5	7.5	17.3
Putamen	14.7	12.0	18.6	9.4	7.8	12.0	13.5	8.1	16.9
Thalamus	15.3	13.2	20.6	13.3	9.5	20.3	15.9	6.4	19.3
Cerebellum	14.9	12.6	17.6	10.5	8.4	14.3	14.1	7.4	18.6
Frontal cortex	14.9	12.9	17.2	10.7	8.1	14.6	14.7	7.3	19.1
Hippocampus	12.8	10.1	14.5	12.1	7.3	21.1	11.5	6.2	14.0
Insular cortex	14.1	12.3	19.0	10.7	8.3	14.2	13.9	7.2	19.8
Lateral temporal cortex	14.2	11.8	17.6	9.8	8.1	13.0	14.1	7.4	17.8
Midbrain	14.4	9.7	22.4	11.0	7.3	18.0	10.9	6.6	13.8
Occipital cortex	14.6	11.9	18.6	10.1	7.4	13.1	14.5	8.0	18.8
Orbitofrontal cortex	19.4	12.0	44.5	10.8	8.3	14.5	14.7	7.8	20.2
Parietal cortex	14.4	12.4	18.2	9.8	7.4	13.3	14.1	7.2	18.5
PCC	16.2	12.7	25.2	11.9	8.0	19.6	15.8	8.4	23.2
Pons	13.7	11.3	17.7	18.0	7.9	38.0	12.9	10.2	15.6

There was no significant difference of the VT values in any region among the three groups.

#### Feasibility of estimation of BP_ND_ and VT for MS lesions

The number of MS lesions detected by MRI for each subject is shown in Table 3. The size of the largest five MS lesions in each subject is shown in Table 3. The size of most MS lesions determined by MRI was below 1 cm^3^. BP_ND_ and VT values estimated by the 2TC model were obtained for the five largest MS lesions of each case (Table 4), but these values were not robust for a single MS lesion on its own. However, even after combining all the MS lesions of a given characteristic (Gd-enhancing or high signal intensity in T2 and/or FLAIR) in each subject to cover a larger area of interest, the calculated VT and BP_ND_ of the combined regions were unrealistically high in some MS lesions and were not robustly obtained (Table 5).

**Table 3:** Number of MS lesions and sizes of the 5 largest MS lesions in each subject

**Subject**	**Number of MS lesions**		**Gd(+)**	**Sizes of five largest MS plaques (cm**^**3**^**)**				
	**Total number**	**T2 and**/**or FLAIR high**		**MSL1**	**MSL2**	**MSL3**	**MSL4**	**MSL5**
A	14	13	2	0.3	0.2	0.1	0.1^a^	0.1
B	20	18	6	6.2	4.3	2.9	1.6	0.7
C	42	42	4	1.3^a^	1.2	1.1	1.0	0.8
D	25	25	3	0.8	0.6	0.6	0.6	0.5
E	31	31	20	0.6	0.6	0.4	0.4	0.2^a^
F	29	29	3	15	4.0	1.7	1.6	1.5
G	10	10	5	10	1.8	0.2^a^	0.1	0.1
H	30	30	3	0.7	0.4	0.4	0.3^a^	0.2
I	10	10	2	0.7^a^	0.3	0.2	0.1	0.1

^a^Gd-enhanced MS lesions. Other values were MS lesions which showed T2 and/or FLAIR high intensity but not Gd-enhanced MS lesions. As some MS lesions show both Gd-enhanced and T2 and/or FLAIR high intensity, the total number of MS lesions is not the sum of T2 and/or FLAIR high-intensity lesions and Gd-enhanced lesions.

**Table 4:** **BP**_**ND **_**and VT values for the five largest MS lesions in 9 MS patients**

**Subject**	**BP**_**ND**_					**VT**				
	**MSL1**	**MSL2**	**MSL3**	**MSL4**	**MSL5**	**MSL1**	**MSL2**	**MSL3**	**MSL4**	**MSL5**
A	-	1.0	-	5.0^a^	4.2	-	1.6	-	17.1^a^	4.4
B	8.2	3.9	5.5	15.7	6.4	15.1	10.6	11.8	13.7	11.1
C	2.4^a^	14.1	5.7	4.2	9.0	12.6^a^	9.4	10.2	15.9	4.8
D	4.3	-	9.3	-	3.3	7.1	-	5.5	-	6.8
E	24.5	-	12.8	9.7	7.9^a^	7.2	-	19.7	11.9	10.2^a^
F	26.9	17.2	3.4	19.4	-	22.9	15.1	8.8	16.5	-
G	4.7	7.7	15.4^a^	0.3	-	11.3	11.6	32.4^a^	8.6	-
H	5.0	-	15.9	26.3^a^	5.4	7.0	6.6	37.5	12.0^a^	5.5
I	12.0^a^	4.0	27.3	16.8	4.0	35.6^a^	30.8	4.6	21.6	6.6

^a^Gd-enhanced MS lesions. Other values were MS lesions which showed T2 and/or FLAIR high intensity but not Gd-enhanced MS lesions.

**Table 5:** **BP**_**ND **_**and VT values of the combined regions in 9 MS patients**

**Subject**	**BP**_**ND**_		**VT**	
	**Gd (+)**	**T2 and/or FLAIR high**	**Gd (+)**	**T2 and/or FLAIR high**
A	12.7	2.6	34.4	4.9
B	26.6	5.7	28.7	11.1
C	5.8	4.5	21.0	9.1
D	3.9	5.9	4.8	7.6
E	5.9	6.3	9.8	13.4
F	12.5	13.3	13.5	13.8
G	3.4	5.0	10.3	11.6
H	60.1	4.6	9.0	9.3
I	11.3	4.0	35.5	12.8

#### Visual inspection of PET images

**Figure 2:**
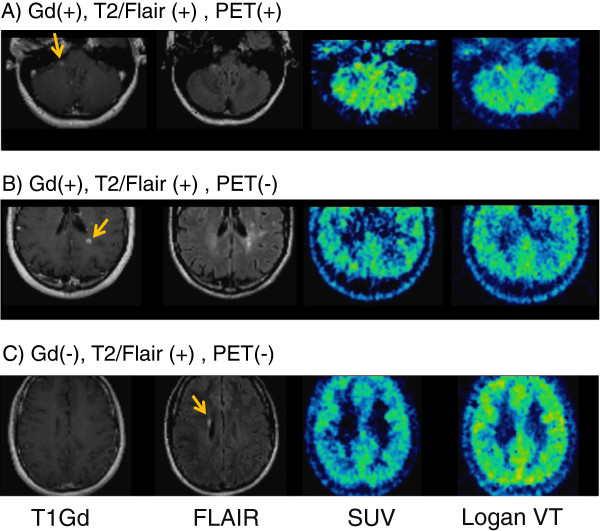
**Typical MRI images and corresponding PET images for MRI**-**defined MS lesions.** There were three patterns as follows: (**A**) Gd-enhanced and high intensity in T2/FLAIR images and high uptake in PET images. The images are from subject A. The arrow indicates the MS lesion. (**B**) Gd-enhanced and high intensity in T2/FLAIR but not high uptake in PET images. The images are from subject D. (**C**) Not Gd-enhanced but high intensity in T2/FLAIR and not high uptake in PET images.

Only one of the Gd-enhanced MS lesions was visually recognized as having higher accumulation when compared to other brain regions in SUV and VT images (Figure 2).

Although some other MRI-defined MS lesions with or without Gd-uptake may also have moderate uptake, this uptake was not clearly higher compared to the surrounding cortical regions. In areas beyond MRI-defined MS lesions, no obvious higher uptake of the radioligand was detected.

### Discussion

#### Kinetic analysis for brain regions

In the present study, we have not detected significant differences in BP_ND_ and VT as outcome measures of [^18^F]FEDAA1106 binding and brain distribution between MS patients and healthy controls in either the whole gray matter or in different brain regions.

There are only limited data available for the imaging of neuroinflammation with TSPO ligands in MS patients, and the patient populations in these studies differ significantly, which makes interpretation and comparison between the studies difficult. Moreover, most of the previous studies did not focus on one clinical subtype of MS but included different forms (e.g., relapsing-remitting as well as secondary progressive diseases), which might confound the results as well.

Initial data obtained with [^11^C]PK11195 on postmortem brain tissue and in a pilot imaging study in two patients showed radioligand binding/uptake and suggested that ligand uptake correlates with the activity of a lesion [11]. An early study with 12 patients using [^11^C]PK11195 showed some increases in focal binding in brains of MS patients and described a certain overlap to signal changes in the MR in single patients [1]; however, the regional analysis in this study was limited as only the brainstem and thalamus have been evaluated. Another PET study with [^11^C]PK11195 in 22 MS patients suggested that ligand uptake was increased in Gd-enhancing lesions compared to normal white matter [2]. However, this study was lacking a more detailed comparison of different brain regions. A more recent PET study using [^11^C]PBR28 as TSPO ligand could not show significant differences with respect to the brain parenchymal binding between healthy volunteers and MS patients. However, contrast-enhancing lesions did show a significantly higher [^11^C]PBR28 binding compared to contralateral NAWM [3].

Although our study focused on relapsing-remitting MS only and specifically aimed at Gd-enhancing lesions, using [^18^F]FEDAA1106 as a new TSPO ligand, we were not able to reproduce these earlier results. This might be due to the specific features of [^18^F]FEDAA1106, which we will discuss in the following in further detail.

The time-activity curves of [^18^F]FEDAA1106 showed slower kinetics in the white matter than in the gray matter, and radioactivity increased during the PET scan time (up to 150 min) in some subjects (data not shown). Due to this, the 2TC model did not converge well. Therefore, white matter might not be an appropriate region for data evaluation using a 2TC model with [^18^F]FEDAA1106.

Another reason possibly obscuring a potential difference of TSPO binding between patients with MS and healthy controls is the ‘non-binder’ issue for TSPO imaging. ‘Non-binders’ have been reported for [^11^C]PBR28 in one out of ten subjects [12]. They showed only very low binding to TSPO. It is reported that there are three different affinity states (high, mixed, and low) for TSPO binding, and it is only recently discovered that genetic polymorphism underlies the binder - non-binder difference [13]. The importance of evaluation of TSPO polymorphisms has been emphasized recently [14-16]. The phenomenon is considered to be common for the newer TSPO radioligands with [^11^C]PK11195 probably being the only exception. Although the existence of different binding states has not been reported for [^18^F]FEDAA1106 yet, it has been reported for [^11^C]DAA1106 [17]. As FEDAA1106 has a similar chemical structure to that of DAA1106, FEDAA1106 is likely to show similar properties and probably will exhibit different binding states as like all newer TSPO ligands including PBR28. There is approximately a 50-fold difference between high and low affinity binders for PBR28 causing the phenomenon of non-binders. For DAA1106, DPA713, and PBR111, the difference between high- and low-affinity binders is about fivefold. The extent of differences between high- and low-affinity binders for FEDAA1106, and its relative impact awaits further study. This information was not available at the time this study was conducted. In future studies, checking the binding state will allow subjects to be stratified into different binding status groups, which might help detect the group differences between patients and healthy controls.

#### Feasibility of estimation of BP_ND_ and VT for MS lesions

BP_ND_ and VT calculated by a 2TC model were not able to quantitatively characterize MS lesions. Previous studies using noise simulation of [^18^F]FEDAA1106 PET data reported that 10% noise-added data generated more than 25% of coefficient of variance in the dorsolateral prefrontal cortex [9]. Considering the small size of the MS lesions, the actual time-activity curves could be much noisier than the aforementioned simulated 10% noise-added data. The unstable estimation of the above outcome measures for MS lesions might be in line with the results from the simulation analysis.

#### Visual inspection of PET images

A previous PET TSPO study using [^11^C]PBR28 showed high uptake in the white matter outside of the MRI-defined MS lesions [3]. Some of the [^11^C]PBR28 high-uptake areas developed to a new Gd-enhanced lesion detected in the 1-month follow-up study. To detect such a change, parametric images could be helpful. Although an approach to de-noise the data in order to improve the parametric images has been reported for [^18^F]FEDAA1106 [18], the BP_ND_ images were not of sufficient quality for visual inspection (i.e., diagnostic practice) due to the low signal-to-noise ratio of [^18^F]FEDAA1106 BP_ND_ PET images.

Although SUV and VT images calculated by the Logan plot were generated in this study, both SUV and VT have some drawbacks for [^18^F]FEDAA1106. SUV values did not always correspond to BP_ND_ or VT values. VT by the Logan plot was reported to be underestimated compared with VT by the 2TC model [9]. In both sets of images, there was no detectable high uptake in the MRI-defined MS lesions, with the exception of one MS lesion (Figure 2). In SUV and VT images, high nonspecific uptake in the surrounding gray and white matter might obscure the changes in the MS lesions and potential changes outside of MS lesions.

The reason why one MS lesion showed high uptake, while other MS lesions did not, could not be fully explained. Although MS lesions might have different features depending on their duration, the time span between the MRI screening and the PET scan of the patient with only MS lesion that showed high uptake was 4 days, and this time span was not different from the average 3.6-day-long time span of all the nine subjects. The MS lesion was located in the right middle cerebellar peduncle close to the brain surface facing the cistern magna. This location could result in less signal spillover and thereby higher contrast to the surrounding tissues. Additionally, lesions, even though they are Gd-enhancing, might still differ with respect to the histological characteristics as well as the respective content and activation of microglia reflected by different amounts of TSPO binding, but this could not be further evaluated in our study.

The PET machine used in this study had a limited spatial resolution, causing most of the MS lesions, which are small, to be affected by a partial volume effect. Higher-resolution PET machines might be an advantage in investigating MS lesions in the future.

## Conclusions

In conclusion, the present study does not endorse the usefulness of [^18^F]FEDAA1106 as a PET radioligand for the differentiation of patients with MS from healthy controls or for the detection of active MS lesions in patients. Even though information on the binding status had been available, the radioligand still showed some unfavorable kinetic characteristics, which would likely hinder the further usage of it in a broader clinical context. ^18^F radioligands with faster kinetics and lower nonspecific binding than [^18^F]FEDAA1106 would have greater potential for the evaluation of patients with MS. Genetic information about TSPO binding must be taken into account in future studies to allow patient stratification.

## Competing interests

TZ, MSM, AT, and AH are employees of Bayer Health Care. VC and CCR are consultants for Bayer Healthcare. The other co-authors have no competing interests.

## Authors’ contributions

AH and CH led the study in total. All authors participated in the development of the study design. FP, JH, and NAT recruited subjects. SN produced the PET radioligand. BG, PS, and AV performed PET measurements. AT (Takano) and AV analyzed the PET image data. TK analyzed the MRI data. All authors contributed to the interpretation of the study results. AT (Takano) drafted the manuscript. All authors critically reviewed the manuscript and approved the final manuscript.
